# Excoriationes Narium

**Published:** 1886-03

**Authors:** Barclay J. Baron

**Affiliations:** Physician to the Bristol General Hospital


### EXCORIATIONES NARIUM. By Barclay J. Baron, M.B., Physician to the Bristol General Hospital.

In the *Monatsschrift für Ohrenheilkunde*, &c., No. 7, 1885, there appears an article bearing the above title, and written by my friend Dr. Schmiegelow, of Copenhagen. Having recently had some cases of the troublesome affection which he describes under my care, in which the ulceration in the outer part of the nostril was progressing, and showed no sign of healing until treated with antibacterial agents, such as carbolic acid lotion, iodoform, &c., I think that the following conclusions which Dr. Schmiegelow has come to, and which I share, are of interest:

1. The term *Eczema narium*, which is sometimes applied to the affection, is misleading, because it only represents the less important and not constant accompaniment of the disease, which is really in the large majority of cases a furunculosis of the sebaceous glands which are connected with the vibrissæ in the nostril. Further, Moldenhauer concludes that the few cases in which there is no furunculosis, and where the affection is a pure eczema, are those of scrofulous children, or of adults where it is an extension of a facial eczema to the nostril.

2. Pasteur and Löwenburg have found a great many micrococci in the pus of a furuncle, and believe that the latter is caused by bacterial infection.

3. Every cause of solution of continuity of the epidermis may produce furuncle, by creating a nidus for the deposit and increase of micrococci, and through their agency inflammation of the sebaceous glands is set up. As chronic eczema of the external auditory meatus is the cause of furuncle in that situation, so eczema of the entrance to the nostril causes furuncle of the nasal cavity, since the patients thus affected, by removing the small scabs that form, produce solution of continuity of the epidermis, by rubbing the part in order to allay the irritation which is present, pull out small hairs, and thus allow of the possibility of micrococci getting into the sebaceous glands and inflaming them.

4. Individuals having numerous stiff vibrissæ in their nostrils are more predisposed to "excoriationes narium" than those who have a few short soft hairs, because the nasal secretion is not collected by the latter to so great an extent as by the former, and therefore scabs are not so readily produced.

5. The affection is most obstinate if it attack the region between the ala and septum nasi, as here there is more chance of stagnation of the nasal secretion, and more difficulty in removing the crusts that form.

6. The aims of treatment are:

1. To prevent the formation and increase of scabs.
2. To protect the epidermis from further solution of continuity.
3. To sterilise the nostril.

7. Epilation is irrational, as it only leads to the opening of new orifices hitherto closed by the hairs, into which micrococci find their way, and by multiplying form abscesses. Baumgarten also denounces this method of treatment. Also the old-fashioned treatment, by means of ointments, glycerine inunctions, and application of nitrate of silver, are all untrustworthy, if not worse.

8. The treatment to be recommended is the following: Small pieces of cotton wool are soaked in an aqueous solution of corrosive sublimate of the strength of 1 to 1,000, or, if this is irritating, of 1 to 2,000. The tampons must be of such a size as to quite fill the nostril, and one nostril at a time is to be filled with its tampon, which must be allowed to remain in it for two hours, then removed, and the other, if affected, operated on. This must be done two or three times daily at first; later on, one tampon a day for each nostril may be sufficient. If irritation is set up by the $HgCl_2$ it should be omitted for a few days, and an ointment, composed of one part of boracic acid to 10 of vaseline, used instead; the use of $HgCl_2$ being resumed when the irritability is allayed. Treated in this way, no new crusts form, the patients do not "pick their noses" and thus increase the area of infection, and the part acted on is sterilised.

9. This treatment has proved most successful in more than forty cases, the cure being absolute in nearly all in the course of a fortnight. If the affection shows any tendency to recur, washing with the weaker solution of $HgCl_2$ is sufficient to prevent it.

10. Where scrofulous children are affected with nasal excoriation, which is often of doubtfully parasitic origin, Kiesselbach recommends the application of tampons which are soaked in a diachylon and petroleum ointment, as the use of $HgCl_2$ for these patients is usually too irritating.

Dr. Schmiegelow has given up the use of the stick of nitrate of silver for these nasal ulcers, and so have I. I may add that in syphilitic and tubercular ulcers of the tongue I have found that the use of a saturated solution of iodoform in ether is very valuable. Numerous cases under my care in which these ulcers had been treated for months by solid caustic, with no effect excepting irritation of the part, yielded to the iodoform treatment, the healing process beginning very soon, and the patients complaining of much less pain after its use. I tell the patients to rinse the mouth with cold water after each meal, and then paint on or spray on to the ulcer some of the iodoform solution, when the iodoform is left in very fine powder on the ulcerated surface.

